# REAC technology modifies pathological neuroinflammation and motor behaviour in an Alzheimer’s disease mouse model

**DOI:** 10.1038/srep35719

**Published:** 2016-10-24

**Authors:** Lorenzini Luca, Giuliani Alessandro, Sivilia Sandra, Baldassarro Vito Antonio, Fernandez Mercedes, Lotti Margotti Matteo, Giardino Luciana, Fontani Vania, Rinaldi Salvatore, Calzà Laura

**Affiliations:** 1 IRET Foundation, Ozzano Emilia, Italy; 2 Department of Veterinary Medical Science, University of Bologna, Italy; 3 Health Science and Technologies Interdepartmental Center for Industrial Research (HST-ICIR). University of Bologna, Italy; 4 Department of Pharmacy and Biotechnology, University of Bologna, Italy; 5 Department of Regenerative Medicine and Neuro Psycho Physical Optimization, Rinaldi Fontani Institute, Florence, Italy; 6 Research Department, Rinaldi Fontani Foundation, Florence, Italy

## Abstract

The search for new therapeutic approaches to Alzheimer disease (AD) is a major goal in medicine and society, also due to the impressive economic and social costs of this disease. In this scenario, biotechnologies play an important role. Here, it is demonstrated that the Radio Electric Asymmetric Conveyer (REAC), an innovative technology platform for neuro- and bio-modulation, used according to the neuro-regenerative protocol (RGN-N), significantly increases astroglial reaction around the amyloid plaques in an AD mouse model, as evaluated by GFAP-immunoreactivity, and reduces microglia-associated neuroinflammation markers, as evaluated by Iba1-immunoreactivity and mRNA expression level of inflammatory cytokines TREM. IL1beta, iNOS and MRC1 were not affected neither by the genotype or by REAC RGN-N treatment. Also observed was an increase in locomotion in treated animals. The study was performed in 24-month-old male Tg2576 mice and age-matching wild-type animals, tested for Y-maze, contextual fear conditioning and locomotion immediately after the end of a specific REAC treatment administered for 15 hours/day for 15 days. These results demonstrated that REAC RGN-N treatment modifies pathological neuroinflammation, and mitigates part of the complex motor behaviour alterations observed in very old Tg2576 mice.

Alzheimer disease (AD) is a major challenge in medicine and society, characterized by no cure, poor symptomatic therapies, and impressive economic and social costs. New therapeutic approaches are needed in this scenario. In recent years, different forms of non-invasive brain stimulation techniques have been applied to patients affected by different neurological and psychiatric disorders, to improve symptoms and possibly slow down disease progression. The most widely investigated technologies include low intensity electromagnetic fields (EMFs) and electric fields. This field is rapidly expanding, and a relatively wide range of stimuli are used in terms of type (magnetic or electric stimulation), and stimulus characteristics (intensity, duration, wave form, stimulation position, therapeutic schema, etc.)^1^. When applied to AD patients, these techniques seem to have beneficial effects on AD-associated cognitive decline^2^.

Numerous non-invasive neuromodulatory techniques other than transcranial magnetic stimulation (TMS) or transcranial direct current stimulation (tDCS) are currently emerging. The so-called second-generation neuromodulation technologies rely on acoustic^3^, electromagnetic induction^4^, optical signals^5^ and wireless deep brain stimulation^6^. The Radio Electric Asymmetric Conveyer (REAC) is a technology that combines low intensity EMFs and electric fields and is based on the production of a flow of electric micro currents in the body of the subject being treated. This current flow can be focused, as required, on specific areas through an (asymmetric) single probe-conveyor, specific to REAC technology. When used according to a specific protocol (neuro-postural optimization –NPO- 250 milliseconds), it induces long-lasting changes in brain activation^7^ and improves balance in patients suffering from various neurological diseases^8^, as observed by functional magnetic resonance imaging (fMRI)^7^,^9^,^10^. The impact of REAC-NPO treatment has also been explored in AD patients in a pilot randomized controlled trial^11^ and in a randomized cross-over trial^12^ designed to investigate the feasibility, safety, and short-term motor effects in patients with advanced AD, who also experience motor deterioration. Both these studies provided promising, albeit preliminary, results. Some tests were conducted also in the treatment of behavioural and psychiatric symptoms in AD^13^.

In order to explore possible mechanisms underlying effects observed after REAC RGN-N treatment in AD patients, the effect was investigated of the specific REAC treatment in a mouse model of AD (Tg2576 mice) tested in very old age (24 months). Tg2576 is a mouse model carrying a single human amyloid precursor protein (hAPP) mutation^14^, and was chosen because of its predictive validity in pharmacological and non pharmacological research^15^, as proven for acetylcholinesterase inhibitors^16^, memantine, galantamine^17^, physical exercise^18^, Ginkgo biloba treatment^19^ and an omega-3 fatty acid enriched diet^20^. An age-dependent impairment in working^21^ and spatial memory^14^, hippocampus-dependent contextual^21^ and amygdala-dependent cued fear learning ability^22^ starting at 5–6 months and persisting across the lifespan^23^ has been described in this mouse. Moreover, while Tg2576 exhibits increased locomotion compared to wild-type (WT) until 14 months of age, locomotion declines in very old mice^24^. Amyloid deposition in the brain starts at 5–7 months, reaching a steady state at 12 months of age^25^. Furthermore, an age-dependent chronic elevation of pro-inflammatory cytokines^26^,^27^ and microglial activation^28^,^29^,^30^,^31^ has been also described in these mice, being neuroinflammation a further hallmark of AD pathology. Neuroinflammation, amyloid pathology and complex behaviours were the exploratory end-points included in this study.

## Results

### Effect of REAC treatment on neuroinflammation

**Figure 1:**
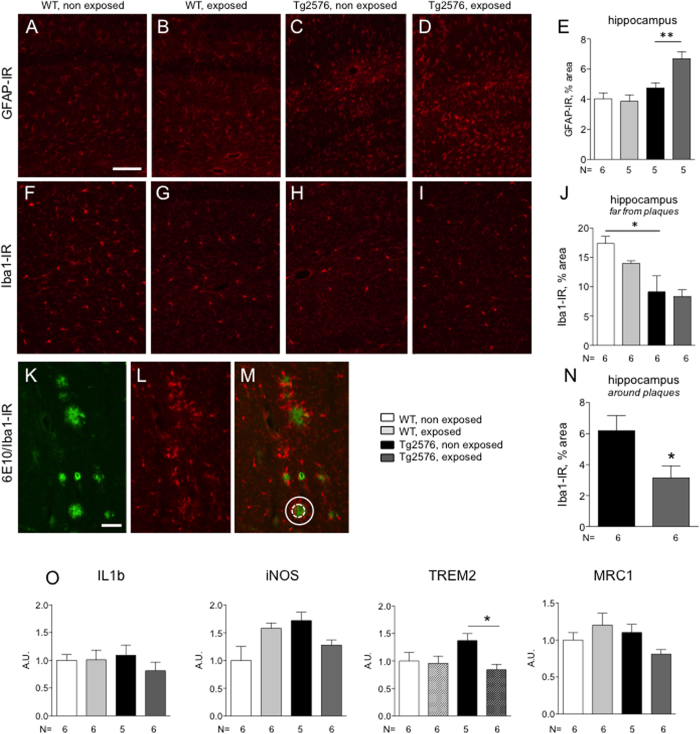
Effect of REAC treatment on neuroinflammation. (**A–D**) representative micrographs and quantification (**E**) of GFAP-IR in the hippocampus of non exposed (**A,C**) and REAC-exposed (**B,D**) WT (**A,B**) and Tg2576 (**C,D**) mice; F-I: representative micrographs and quantification (**E**) of Iba1-IR in the hippocampus (far from plaques) of non exposed (**F,H**) and REAC-exposed (**G,I**) WT (**F,G**) and Tg2576 (**H,I**) mice; (**K–M**): representative micrographs of 6E10-IR amyloid plaques (green, **K**), Iba1-IR (red, **L**) and merged image (**M**) in the hippocampus of Tg2576 mice. The circles in M refers to the plaque diameter (dotted line) and the 2x surrounding areas included in the Iba1-IR analysis (continuous line). N: quantification of Iba1-IR around the plaques. Data are expressed as mean + SEM. Statistical analysis was performed by two-ways ANOVA and post-hoc test. (*P < 0.05; **P < 0.01). Bars: A (AD; (**F–I**): 20 μm; K (**L,M**): 50 μm. O. Effect of REAC treatment on cytokine synthesis. mRNA expression level of IL1beta, iNOS, TREM and MRC1 in the hippocampus of WT and Tg2576 mice, non exposed and exposed to REAC are shown. Arbitrary Units (A.U.) indicate the relative mRNA quantity normalized to WT non exposed group. The number of animals included in each assay is indicated below the respective graph bar; data are expressed as mean + SEM. Statistical analysis was performed by two ways ANOVA and post-hoc test (*P < 0.05).

As neuroinflammation is a primary hallmark of AD, the effect of REAC treatment on inflammatory cells in the brain (astrocyte and microglia) and cytokines synthesis were investigated. Astrocytes were labelled using an antibody targeting the glial fibrillary acid protein (GFAP). GFAP immunoreactivity in representative hippocampal sections of WT and Tg2576 are shown in Fig. 1A–D, where A and C are from non-exposed, and B and D are from REAC-exposed mice. Quantitation of immunostaining in the hippocampus showed that REAC exposure increases GFAP-IR in Tg2576 but not in WT animals (Fig. 1E, P < 0.01) (two-ways ANOVA: genotype F(1,17) = 18.32 P = 0.0005; treatment F(1,17) = 4.618 P = 0.0463; interaction F(1,17) = 6.594 P = 0.0200).

Microglia was investigated with the use of an antibody targeting the glia-specific calcium-binding adaptor protein Iba1, and analysed far and close to the amyloid plaques. Iba1 immunoreactivity in representative hippocampal sections of WT and Tg2576 are shown in Fig. 1F–I and H,I, where F and H are from non-exposed, and G and I are from REAC-exposed mice. Quantitation of immunostaining in the hippocampus revealed a lower expression level in Tg2576 mice, when measured far from the plaques (Fig. 1J, P < 0.01), while REAC treatment does not modify ionized calcium-binding adapter molecule 1 (Iba1)-IR either in WT and TG2576 mice (two-ways ANOVA: genotype F(1,20) = 19.57 P = 0.0003; treatment F(1,20) = 1.382 P = 0.2536; interaction F(1,20) = 2.598 P = 0.1227).

We also analysed the Iba1-IR around the plaques (i.e. covering an area with 2x the plaque diameter, see Imbimbo *et al*., 2009), as illustrated in Fig. 1K–M, where 6E10-IR plaques are in green (K), Iba1-IR in red (L), merged image is in M, and quantification in N. REAC treatment induces a significant reduction in Iba1-IR around the plaques (p = 0.0209).

In view of the above results, the mRNAs expression level of some cytokines associated with microglia activation was investigated. Results are presented in Fig. 1O. No changes were observed in interleukin 1beta (IL1beta), iNOS, and Mannose Receptor, C Type 1 (MRC1) mRNA expression level (two-ways ANOVA: IL1beta genotype F(1,19) = 20.39 P = 0.6567; treatment F(1,19) = 0.8609 P = 0.3651; interaction F(1,19) = 1.031 P = 0.3226; iNOS genotype F(1,19) = 4.262 P = 0.0529; treatment F(1,19) = 0.1240 P = 0.1443; interaction F(1,19) = 12.00 P = 0.0026; MRC1 genotype F(1,19) = 1.850 P = 0.1897; treatment F(1,19) = 0.3629 P = 0.5540; interaction F(1,19) = 5.302 P = 0.0328). REAC treatment induces a significant reduction in the mRNA expression level of Triggering Receptor Expressed On Myeloid Cells 2 (TREM2) (P < 0.05) in Tg2576, but not WT mice; TREM2 (two-ways ANOVA: genotype F(1,19) = 0.4697 P = 0.5014; treatment F(1,19) = 4.615 P = 0.0448); interaction F(1,19) = 3.447 P = 0.0789).

### Effect of REAC treatment on complex behaviour

**Figure 2:**
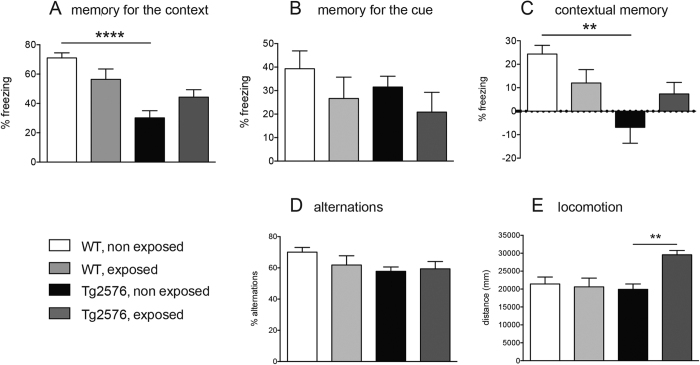
Effect of REAC treatment on learning, memory and exploratory locomotion, as evaluated by contextual fear conditioning % freezing Context-% Freezing baseline Cue (**A–C**) and Y-maze (**D,E**). (**A**) memory for the context (**B**) the memory for the cue; (**C**) contextual memory. (**D**) % of alternations, calculated as (N° of total alternations/total arms entry-2)*100 over 8 min trial. (**E**) travelled distance for the entire duration of the test. Six animals × group were included in these experiments; data are expressed as mean + SEM. Statistical analysis was performed by two ways ANOVA and post-hoc test (*P < 0.05; **P < 0.01).

To establish whether the gliosis regulation by REAC impacts on complex behaviour, the effect of REAC treatment on learning and memory tasks, gait and locomotion was examined. Contextual fear conditioning (CFC) measures the ability of the mouse to learn and link a distinct context with aversive foot shocks, thus exploring associative learning ability. Results of the CFC test in REAC exposed and not exposed WT and Tg2576 mice are reported in Fig. 2. Mean (±SEM) percentage of freezing during the conditioning context (old context), after the conditioning sound in the new environment (cue) of the test session (Day 2), and the derived contextual memory are reported in A, B, and C, respectively. During the test day, Tg2576 mice displayed no differences in the cue phase of the test, and a significantly lower freezing behaviour in the old context (P < 0.001) and contextual memory (P < 0.01) compared to wild-type animals (two-ways ANOVA: old context, genotype F(1,20) = 18.63 P = 0.0003; treatment F(1,20) = 0.2633 P = 0.6135; interaction F(1,20) = 9.478 P = 0.0059; contextual memory, genotype F(1,20) = 9.994 P = 0.0049; treatment F(1,20) = 0.0022 P = 0.9628; interaction F(1,20) = 7.403 P = 0.0132). REAC treatment had no effect.

Animals were also tested in Y-maze (Fig. 2D,E). Y-maze spontaneous alternation is a behavioural test for measuring the willingness of rodents to explore new environments. No differences between WT and Tg2576 mice, and between exposed and not-exposed mice were observed in Y-maze alternation (D) (two-ways ANOVA: genotype F(1,20) = 2.196 P = 0.1540; treatment F(1,20) = 0.7653 P = 0.3921; interaction F(1,20) = 0.02582 P = 0.8740). However, REAC treatment increases the distance travelled over the 8 min test in Tg2576 (p < 0.05), but not WT mice (E) (two-ways ANOVA: genotype F(1,20) = 10.34 P = 0.0043; treatment F(1,20) = 17.09 P = 0.0005).

### Effect of REAC treatment on amyloid pathology

**Figure 3:**
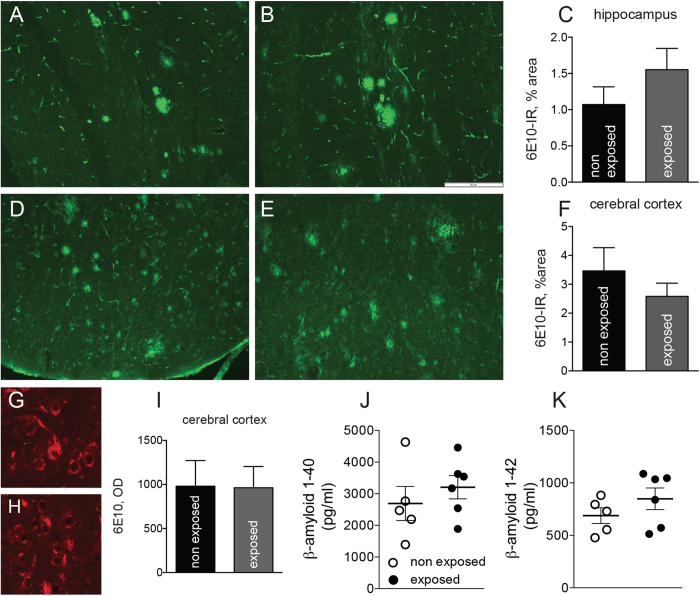
Effect of REAC exposure on brain amyloid pathology. (**A,B**) representative micrographs (**A,B**) and quantification (**C**) of amyloid plaque burden in the hippocampus of non exposed (**A**) and REAC-exposed (**B**) Tg2576 mice; (**D,E**) representative micrographs (**D,E**) and quantification (**F**) of amyloid plaque burden in the cerebral cortex of non exposed (**D**) and REAC-exposed (**E**) Tg2576 mice; (**G**,**H**) representative micrographs and quantification (**I**) of intraneuronal 6E10-IR amyloid in the cerebral cortex of non exposed (**G**) and REAC-exposed (**H**) Tg2576 mice. Bar: 20 μm. (**J**,**K**) Amyloid 40 (**J**) and 42 peptide (**K**) in the plasma of non exposed (white dot) and REAC-exposed (black dot) Tg2576 mice. Six animals in the “exposed” and five animals in the “non exposed” groups were included (see methods for details). Data are expressed as mean + SEM. Statistical analysis was performed by Student’s t test.

Amyloid plaque burden was visualized with the anti-amyloid β1–16 monoclonal antibody (mAb) 6E10 in the cerebral cortex (Fig. 3A,B) and hippocampus (Fig. 3D,E), and semi-quantitatively determined by digital image analysis. This antibody reacts with the soluble Amyloid Precursor Protein (sAPP) β precursor as well as with the processed forms of amyloid β peptides. Results are presented in Fig. 3C (hippocampus) and F (cerebral cortex). REAC exposure does not modify amyloid plaque burden in Tg2576 mice. Intraneuronal Aβ (APP) levels were also investigated. Representative images of brain cortex specimens derived from unexposed (panel G) and REAC exposed (panel H) mice are shown in Fig. 3. No differences were observed between exposed and unexposed mice.

Finally, the amyloid peptide beta 40 (Ab40) and Ab42 plasma levels were measured in Tg2576 mice (Fig. 3J,K). All samples analyzed showed values within the dynamic range of the standard curve for β40 and Aβ42. The plasma levels of Aβ40 and Aβ42 peptides were not significantly different in control Tg2576 compared with REAC exposed mice.

## Discussion

Previous studies^11^,^12^ conducted in humans with advanced AD, showed that REAC–NPO administration improves axial movement. Accordingly, it was decided to use 24month-old Tg2576 mice, i.e. in a severe, no longer evolving stage of the pathology, and locomotion was included as end-point.

The most significant effect of REAC treatment is the regulation of some of the inflammatory markers in Tg2576, but not WT mice. REAC treatment increases astrocyte-associated GFAP-IR. In the AD brain, reactive astrocytes surround plaques, particularly dense-core plaques, which are those mainly found in Tg2576 mice^32^. *In vitro* and *in vivo* studies indicate that astrocytes might play a role in the removal of Aβ deposits^33^. In APP/PS1 transgenic mice, astrocytes exhibit a significant increase in spontaneous activity^34^ and in the Tg2576 transgenic mouse astrocytes seem to exert a protective and defensive response to fibrillar Aβ^35^. It was also found that an astroglial hyperactivity is associated with positive effects of drugs on learning, memory and synaptic pathology in Tg2576 mice, as for a nonsteroidal anti-inflammatory derivative^36^,^37^.

REAC treatment also decreases some microglia markers, as indicated by Iba1-IR and mRNAs expression levels for TREM mRNAs. Microglia modulation has actually been identified as a primary therapeutic target in AD^38^. Depending on their activation status and the pathologic events encountered, microglial cells are able to exert a variety of functions, which may be either neurotoxic or neuroprotective. The classic paradigm for microglia action in AD involves microglial accumulation around plaques. Although microglia have the capacity to remove β-amyloid deposits and alleviate disease pathology, they fail to do so as the disease progresses. Instead, they become chronically activated, promoting an inflammation-mediated impairment of cognition and cytotoxicity^33^. REAC treatment decreases Iba1-IR around amyloid plaques, and also decreases TREM2 mRNA expression level. Iba1-IR cells surrounding amyloid plaques was indicated as a marker for microglial activation in Tg2576 mice^39^. In the AD brain and in mouse models of AD, plaque-associated cells express high levels of TREM2, a triggering receptor expressed on myeloid cells 2, whose mutations have been found in AD patients^40^, that also increases with age and disease progression^41^. iNOS and MRC1 are associated to microglia type 1 and 2, respectively. However, both iNOS and MRC1 regulation is strongly age-dependent^42^,^43^. The effect of REAC treatment on different neuroinflammation markers in very old AD mice is remarkable, although fuzzy, in view of the role attributed to neuroinflammation in the pathogenesis and progression of AD, no longer as an ancillary event driven by amyloid plaques deposition, but as a major player^44^,^45^. Notably, an age-dependent increase of neuroinflammation and microglia is described in Tg2576 mice^26^,^27^,^28^,^29^.

A correlation between REAC effects on neuroinflammation and complex behaviour is not possible from this study. REAC treatment improved locomotion over 8 min in very old Tg2576, when the animals are no longer hyperactive, but not in WT mice, as measured in the exploratory set-up of the Y-Maze, whereas had no effect on learning and memory performance.

As regards the REAC treatment efficacy on amyloid pathology, no effects are observed at this stage of the disease. This is not surprising, considering that in Tg2576 mouse plaques are fixed starting from 18 months, and neither new plaques nor growth of pre-existing plaques are observed over 6 weeks, as indicated by two-photon *in vivo* microscopy^46^. On the contrary, in 12 month-old Tg2576 mice a turnover in amyloid plaques is observed.

In conclusion, in this proof-of-concept study the administration of the RGN-N protocol of the REAC technology platform was observed to produce a general decrease in neuroinflammation markers in very old Tg2576 mice and an increase in exploratory locomotion. Further experiments will test REAC efficacy in large groups of Tg2576 and WT mice, in particular to establish if REAC treatment could positively modulate early neuroinflammatory events and synaptic pathology occurring before plaque deposition. Although these results must be considered as still only preliminary, to the best of our knowledge this is the first report describing a modulation of neuroinflammation in AD animal models by neurostimulatory/modulatory techniques.

## Material and Methods

### Ethics

All the animal protocols described here were carried out in compliance with the European Community Council Directives of 24 November 1986 (86/609/EEC) and approved by intramural committee of Bologna University and by Ministero Salute, in accordance with the guidelines published in the Guide for the Care and Use of Laboratory Animals and ARRIVE guidelines.

### Animals, genotyping, and sacrifice

Tg2576 transgenic mice carrying a transgene coding for the 695-amino acid isoform of human amyloid precursor protein derived from a large Swedish family with early-onset AD^14^ were used in this study (Taconic Europe, Lille Skensved, Denmark). Mice tails were used for genotyping analysis. The mice genomic DNA was extracted using the GenEluteTM Mammalian Genomic DNA MiniPrep Kit (SIGMA) according to the instructions of the manufacturer, and eluted in 100 μl of elution solution. DNA concentration was determined using a spectrophotometer and Tg2576 mice were identified by the presence of the mutated human APP gene by Real Time PCR technique, using the MaximaR Sybr Green/Rox qPCR Master Mix (Thermo Scientific, Fermentas). The amount of DNA used for each sample was 0.5 μg and PCR amplification conditions were: 62 °C for 8 seconds, followed by an extension step at 72 °C for 20 seconds (FW: 5′–GATGAGGATGGTGATGAGGTA-3′ REV: 5′–ACTGGCTGCTGTTGTAGG–3′). Only the animals expressing the mutated human amyloid precursor protein gene have been used for the experiments. All animals were negative for the retinal degeneration Pde6b(rd1) (rd) mutation.

Twenty-four-months-old transgenic male (N = 12) and aged-matched non-transgenic male littermates (N = 12) were used. Animals from each genotype were randomly assigned to the treatment groups, and the final group composition was the following: WT, non exposed, N = 6; WT, REAC-exposed, N = 6; Tg2576, non exposed, N = 6; Tg2576, REAC-exposed, N = 6. However, one mouse in Tg2576 non exposed group died after behavioural tests. Due to the different post-mortem stability of the investigated antigens and the mRNAs, this mouse was then used for IHC experiments, but not for mRNA analysis and amyloid peptides plasma assays. The number of the animals included in each assay is indicated in the figures and/or in the legend to the figure.

Animals were exposed to REAC RGN-N for 12 hour/day (dark phase) for 15 days in single polypropylene cage (16 cm × 20 cm), having an aluminium floor partially covered by wood litter (see Fig. 1 in Supplementary Materials). Non-exposed animals were housed in the same conditions as exposed animals, just excluding exposure.

At the time of sacrifice, in deeply anesthetized animals, blood was collected from descending aorta; half-brain in each animal was dedicated to morphological experiments (immunohistochemistry), half-brain to molecular biology (RT-PCR).

### Description of Radio Electric Asymmetric Conveyer (REAC) Technology

Radio Electric Asymmetric Conveyer Technology (REAC) is a technology platform for bio- and neuro-modulation. The purpose of REAC technology is to optimize the ion fluxes at the molecular level, and to concentrate all the micro currents produced by these ion fluxes through the asymmetric conveyer-probe (ACP). The radio frequencies interact with all the structures that contain electrical charges, such as the human body, and induce currents in them. These currents vary according to the molecular characteristics of the tissues. The REAC device used in this study was set for the regenerative type N protocol (RGN-N) to generate a 2.4 GHz radiofrequency emission of very low intensity (0.1 V/m at a distance of 3 m from the emitter), with on/off waveform modulation (750 ms on/1500 ms off). The peculiarity of REAC technology is not the emission itself, but the particular physic link between the device and the body. The “Asymmetric Conveyer Probe” (ACP) represents this link. This is the innovation of REAC technology^47^,^48^. The stimulation position is represented by the point of the body where the ACP is positioned. In this study, the ACP was the aluminum cage floor, while the stimulation points were the mice paws. Postural position has no influence on REAC treatment effects, but on the contrary, several previous studies have proven that REAC treatments can improve postural position and motor behaviour^7^,^8^,^9^,^10^,^11^,^12^. Therefore, there is no need to control mice position during the treatment. The REAC model device used in this study was B.E.N.E. (ASMED, Florence, Italy)^49^,^50^,^51^,^52^.

### Behavioural analysis

Animals were tested for learning and memory by two different tests. Spatial memory was evaluated by the Y-maze test 24 hours after the last REAC treatment. Spontaneous alternation under spontaneous behavioral conditions was evaluated using Y-Maze test. Mice were tested in dark gray maze (35 cm long × 10 cm high × 5 cm wide; Ugo Basile, Comerio, Italy). The room lighting is set so that the center of the arena there are 60 ± 5 Lux. Each mouse was placed at the end of one of the arms and allowed to move freely through the maze for an 8‐min test session. The sequence of arm entries was recorded by the videotracking software AnyMaze (Stoelting, Wood Dale, IL, USA). Arm entry was defined as entry of all four paws into one arm. A correct alternation occurred when the animal moved to the other two arms without retracing its steps (i.e. Arm A to B to C). Movements such as ABA were incorrect. The percentage of correct alternations was calculated by the following formula: {n°/(total arm entries − 2)} × 100. Animals that did not perform at least 10 entries were excluded by the analysis.

The memory for the context was tested in the contextual fear conditioning (CFC) 48 hours after Y-maze test. Mice were trained and tested on 2 consecutive days. The test was performed in 30 × 24 × 21 cm operant chambers provided with one house light; a speaker and a web-cam are placed inside (Ugo Basile, Comerio, Italy). Training consisted of allowing the subject to explore the operant chamber for 2 min, afterward an auditory cue [2000 Hz, 50 dB; conditioned stimulus (CS)] was presented for 15 s. The footshock [0.6 mAmp; unconditioned stimulus (US)] was administered for the final 2 s of the CS. This procedure was repeated, and mice were removed from the chamber 1 min later. Twenty-four hours after training, mice were returned to the same chambers in which training occurred and freezing behaviour was recorded by the software. Freezing was defined as lack of movement except that required for breathing, and described as “memory for the context”. At the end of the 5 min, mice were returned to their home cage. At least 1 h later, freezing was recorded in a novel environment and in response to the cue, and described as “memory for the cue”. The novel environment consisted of modifications of the operant chamber including a grey Plexiglas divider bisecting the chamber, a Plexiglas floor, and decreased illumination. Mice were placed in the novel environment without any stimulus and freezing was scored for 3 min. The auditory cue (i.e. CS) was then presented for 3 min, and freezing was again scored. The memory for the cue was calculated as % of freezing of the sound (cue stage) − % freezing Baseline (cue stage). In order to exclude bias due to spontaneous movement, the “contextual memory” was also calculated as follow: % freezing (old context) − % freezing baseline (cue stage)^53^.

Mice activity in both tests was recorded by ANY-Maze video-tracking software (Stoelting). Both systems were provided by UGO BASILE (Comerio Verese, Italy).

### Total RNA isolation, reverse transcription and semi-quantitative real-time PCR

Hippocampi were homogenized and total RNA isolation was performed using InviTrap^®^ Spin Universal RNA Mini Kit (Stratec Molecular). Total RNA was eluted in RNase Free Water and using a spectrophotometer (Nanodrop 2000, Thermo Scientific), absorbance values at 260, 280 and 320 were measured.

RNAs were subjected to DNase treatment (1 U/μl, 1x DNase buffer, 2 U/μl ribonuclease inhibitor, at 37 °C for 30 min) (Fermentas, Life Sciences, Italy) and were retrotranscribed using the enzyme M-Moloney murine leukaemia virus reverse transcriptase (M-MuLV-RT, 10 U/μl) (Fermentas), in the presence of 1x first strand buffer, 1 mM d(NTP)s (Fermentas), 25 ng/μl Oligo (dT)18 primers (Fermentas) by incubating at 42 °C for 60 min. A sample with no reverse transcriptase enzyme in the reaction mix was processed as no RT control.

Semi-quantitative real-time PCR was performed using the Mx3005PTM real-time PCR system (Stratagene, CA, USA). Specific primers pairs were used for each analysis (Table 1). The chemistry chosen to perform these PCR experiments was based on SYBR Green I fluorescent detection and each reaction mix consisted of 10 ng of template cDNA, 1x Maxima™ SYBR Green/ROX qPCR Master Mix (Fermentas) and 0.4 μM of both primers (sense and antisense). The sequences of specific primers for each gene of interest were reported in Table 1.

Among these, GAPDH was considered as housekeeping gene. All primers were obtained from IDT (Coralville, IA, USA). In order to check for possible contamination of genomic DNA, the RT control was processed. No template controls were used for each reaction mix.

Thermal profile of PCR reactions was performed as follow: an activation step of master mix Taq polymerase (95 °C, 10 min) and 40 cycles of denaturation (95 °C, 15 sec) and annealing/extension (60 °C for 30 sec). At the end of the amplification cycles the dissociation curve was obtained by following a procedure consisting of first incubating samples at 95 °C for 1 min to denature the PCR-amplified products, then ramping temperature down to 55 °C and finally increasing temperature from 55 °C to 95 °C at the rate of 0.2 °C/s, continuously collecting fluorescence intensity over the temperature ramp.

The specificity of the amplified product was verified by the presence of a single peak at the expected melting temperature. Random amplified products were resolved by electrophoresis in a 2.0% agarose gel stained with ethidium bromide in order to check the specificity of the PCR reaction. This was confirmed by the presence of a single band of the expected size. A 100 bp DNA ladder (Fermentas) was used as DNA marker. The semi-quantitative analysis of gene expression was performed on the values of the threshold cycle (Ct) obtained for each sample, considering GAPDH as housekeeping gene. Samples were always processed in duplicate. The relative gene expression was calculated with the formula 2^(−ΔΔCt)^ using a defined group as reference (2 ^(−ΔΔCt)^ = 1).

### Immunofluorescence and image analysis

After sacrifice, half-brain from each animal was removed and immersed for 24 h in the ice-cold fixative (4% paraformaldehyde and 14% picric acid saturated solution in Sorensen phosphate buffer 0.2 M pH 7), then rinsed for at least 48 h in 5% sucrose in 0.1 M phosphate buffer. Brains were frozen in CO2 and 14 micron thick sagittal sections were then collected, from 1.8 mm level according to Paxinos and Franklin^54^, on gelatin coated slides using a cryostat (HM550 Microm).

Sections were first incubated in 0.1 M phosphate buffered saline (PBS) at room temperature for 10–30 min, followed by overnight incubation at 4 °C in a humid atmosphere with the primary antibodies diluted in 0.3% PBS-Triton X-100: mouse monoclonal anti-6E10 (1:1000, Covance); rabbit polycolan anti-GFAP (1:500, Dako) and rabbit polycolan anti-Iba1 (1:500, Wako). After rinsing in PBS for 20 min (2 × 10 min), the sections were incubated at 37 °C for 30 min in a humid atmosphere with the secondary antisera conjugated with different fluorochromes: DyLight488-conjugated affinity-pure Goat anti-Mouse IgG (ThermoScientific), Rhodamine Red™-X-conjugated -conjugated affinity-pure Donkey anti-Rabbit IgG (Jackson Immunoresearch) diluted in PBS triton 0.3%. Sections were then rinsed in PBS (as above) and mounted in glycerol containing 1,4-phenylendiamine (0.1 g/l).

Control slices incubated with only secondary antibodies were processed in parallel. In order to minimize technical bias, the following technical features were taken: all sections to be compared were processed in the same experimental session; 6 brains from different experimental groups were included in each slide, according to a random distribution; the same batch, dilution and preparation of primary such as secondary antibodies were used for all sections to be compared; the same conditions were established for all sections.

Images were captured using a Nikon Eclipse E600 microscope equipped with digital CCD camera Q Imaging Retiga-2000RV (Q Imaging, Surrey, BC, Canada). Measurements were performed using Nis-Elements AR 3.2 software. The immunoreactive area was calculated as % area of the region of interest (ROI). The mean intensity was expressed as optical density (OD) of the immunoreactive signal, subtracted of the background. For the analysis of intraneuronal Aβ immunostaining, the optical density in single cells in about 50 neurons in the anterior cingulated cortex were measured in each animal. For the analysis of amyloid plaques (6E10-), GFAP- and Iba1-immunoreactivity (IR), twelve counts were performed for each animal. Analyses were performed in analogous areas of the cortex (somatosensory cortex) and hippocampus (across the dentate gyrus) using a 10x objective, using the automated target detection mode. Image size was 1280 × 960 pixels with a target area size of 68,000 μm^2^, and the immunoreactive area/total area (%area) was calculated.

For all markers, the mean value/animal was used for statistical analysis. All analyses were performed in a blinded manner. The sections in which the ratio between IR and background autofluorescence, as evaluated by control sections incubated with the secondary antibody, only, was <2 were excluded from the analysis.

### Aβ40 and Aβ42 plasma levels

Blood samples were collected in EDTA-vacuum collection tubes, centrifuged at 4000 g for 10 min and plasma aliquots were stored at −80 °C. Simultaneous quantification of β-amyloid1-40 (Aβ40) and β-amyloid1-42 (Aβ42) peptides was performed on properly diluted plasma samples using the INNO-BIA plasma Aβ forms kit (Innogenetics NV, Gent, Belgium, through Innogenetics srl, Italy), following the manufacturer’s instructions. This kit is a fluorimetric bead-based immunoassay using Luminex xMAP^®^ technology. Briefly, the Aβ40 and Aβ42 peptides are captured selectively by a mix of beads (xMAP^®^ microspheres) coated with three monoclonal antibodies (mAb): 21F12 for Aβ42, 2G3 for Aβ40 and AT120 for matrix. Following an overnight incubation step at 4 °C, the mix is washed and subsequently incubated with the “detection conjugate” solution (phycoerythrin-labeled streptavidin) at room temperature for 1 h. The mix of beads is then washed and read using the Luminex 200™ IS Total System, which analyzes the microspheres/beads in a flow stream. The fluorescence signals associated with individual beads are converted into intensity units by a digital signal processor and then related to the concentration of the bound antigen through the Xponent 3.1 software.

### Statistical analysis

Data from single animals were the unity of analysis in all experiments. The number of animals included in each assay is indicated in the figure or in the figure legend. The statistical analysis was performed using the GraphPad Prism software (version 6). Each data set was analysed using two-way ANOVA followed by Bonferroni’s post-hoc tests for exposure and genotype. Behavioral experiments were analysed using two-way ANOVA followed by Sidak’s post-hoc tests for exposure and genotype. Post tests were applied only for statistically significant two-way ANOVA interactions (P < 0.05). Student’s t test was used for two-groups comparison. A probability level of P < 0.05 was considered to be statistically significant.

## Additional Information

**How to cite this article**: Luca, L. *et al*. REAC technology modifies pathological neuroinflammation and motor behaviour in an Alzheimer’s disease mouse model. *Sci. Rep.* **6**, 35719; doi: 10.1038/srep35719 (2016).

## Figures and Tables

**Table 1:** Sequence of specific primers used in the study for PCR experiments.

GENE	REVERSE PRIMER	FORWARD PRIMER
**IL1beta**	5′-GCA GGG TGG GTG TGC CGT C-3'	5′-AAA GCC TCG TGC TGT CGG ACC-3'
**iNOS**	5′TCGATGCACAACTGGGTGAA-3'	5′-GCCACCAACAATGGCAACAT-3'
**TRΕΜ2**	5′-AAAGTACTGGTGGAGGTGCTG-3'	5′-TCTTGATTCCTGGAGGTGCTG-3'
**MRC1**	5′-TGGTTCACCGTAAGCCCAAT-3	5′-GGCTGATTACGAGCAGTGGAA-3'
**GAPDH**	5′-ACATACTCAGCACCAGCATCACC-3'	5′-GGCAAGTTCAATGGCACAGTCAAG-3'
