# Erratum: REAC technology modifies pathological neuroinflammation and motor behaviour in an Alzheimer’s disease mouse model

**DOI:** 10.1038/srep37483

**Published:** 2016-11-18

**Authors:** Luca Lorenzini, Alessandro Giuliani, Sandra Sivilia, Vito Antonio Baldassarro, Mercedes Fernandez, Matteo Lotti Margotti, Luciana Giardino, Vania Fontani, Salvatore Rinaldi, Laura Calzà

Scientific Reports 6: Article number: 3571910.1038/srep35719; published online: 10 24 2016; updated: 11 18 2016.

The original version of this Article contained errors in the spelling of the authors Luca Lorenzini, Alessandro Giuliani, Sandra Sivilia, Vito Antonio Baldassarro, Mercedes Fernandez, Matteo Lotti Margotti, Luciana Giardino, Vania Fontani, Salvatore Rinaldi & Laura Calzà which were incorrectly given as Lorenzini Luca, Giuliani Alessandro, Sivilia Sandra, Baldassarro Vito Antonio, Fernandez Mercedes, Lotti Margotti Matteo, Giardino Luciana, Fontani Vania, Rinaldi Salvatore & Calzà Laura respectively.

The Author Contributions Statement,

L.L.: study design, animal care and REAC exposur G.A.: behavioural testing S.S.: immunohistochemistry and image analysis B.V.A.: real time PCR F.M.: amyloid peptide assay L.M.M.: statistical analysis G.L.: study design, manuscript preparation F.V.: REAC technology advice, manuscript preparation R.S.: REAC technology advice, manuscript preparation C.L.: study design, data analysis, manuscript preparation.

now reads:

L.L.: study design, animal care and REAC exposur A.G.: behavioural testing S.S.: immunohistochemistry and image analysis V.A.B.: real time PCR M.F.: amyloid peptide assay M.L.M.: statistical analysis L.G.: study design, manuscript preparation V.F.: REAC technology advice, manuscript preparation S.R.: REAC technology advice, manuscript preparation L.C.: study design, data analysis, manuscript preparation.

The Competing Financial Interests statement,

L.L., G.A., S.S., B.V.A., F.M., G.L. and C.L. have no competing financial interests to declare. R.S. and F.V. are the inventors of REAC Technology.

now reads:

L.L., A.G., S.S., V.A.B., M.F., M.L.M., L.G. and L.C. have no competing financial interests to declare. S.R. and V.F. are the inventors of REAC Technology.

In addition, the ‘How to cite this article’ section quoted an incorrect abbreviation for Luca Lorenzini.

These errors have now been corrected in the PDF and HTML versions of the Article.

